# Sustainable nanophytosome-based therapies against multidrug-resistant *Escherichia coli* in urinary tract infections: an in Vitro and in vivo study

**DOI:** 10.1186/s12951-024-03006-1

**Published:** 2025-03-06

**Authors:** Ming Ming Wen, Ibrahim A. Abdelwahab, Rania Abozahra, Sarah M. Abdelhamid, Kholoud Baraka, Heba Essam Sedky Ahmed, Wessam F. El-Hadidy

**Affiliations:** 1 Department of Pharmaceutics & Pharmaceutical Technology, Faculty of Pharmacy, Pharos University in Alexandria, Alexandria, Egypt; 2 Department of Microbiology and Immunology, Faculty of Pharmacy, Pharos University in Alexandria, Alexandria, Egypt; 3 Department of Microbiology and Immunology, Faculty of Pharmacy, Damanhour University, Damanhour, Egypt; 4 Department of Pharmacology & Experimental Therapeutics, Medical Research Institute, Alexandria University, Alexandria, Egypt

**Keywords:** Nanophytosomes, Rosemary oil, *Escherichia coli*, Chitosan, Urinary tract infection, Multidrug resistance, Drug delivery, Sustainability, Herbal medicine, Nanoparticle

## Abstract

**Background:**

Urinary tract infection (UTI) is a prevalent bacterial infection impacting a significant number of individuals globally. The rise in multidrug-resistant (MDR) *E. coli* strains as the predominant cause of UTIs presents a substantial public health concern and poses a challenge to existing antibiotic treatments. This study introduces an innovative and sustainable therapeutic approach utilizing rosemary oil nanophytosomes as a targeted drug delivery system to address biofilms in UTIs induced by MDR *E. coli*.

**Method:**

Seventy clinically identified *E. coli* isolates from UTI patients were used for this study. Nanophytosomes were formulated with chitosan (CS) and nanostructured lipid carriers. CS-nanophytosomes were lyophilized to evaluate the storage stability. In vivo study included 40 female Wistar rats with daily treatment over seven days. For all the statistical tests, differences were considered significant at *p* < 0.01 and highly significant at *p* < 0.001.

**Results:**

CS-nanophytosomes demonstrated a particle size of 176.70 ± 12.30 nm with a substantial antibiofilm efficacy against MDR *E. coli*. High entrapment efficiency was ascertained with 93.12 ± 1.05%. The drug release study showed that the pure rosemary oil exhibited a notably lower release of 35.4 ± 2.36% over 48 h. In contrast, the CS-nanophytosomes and lyophilized CS-nanophytosomes displayed significantly higher release percentages of 58.6 ± 3.69% and 56.9 ± 5.01%, respectively, compared to the pure rosemary oil of 35.4 ± 2.36% over 48 h. The in vivo study indicated that nanophytosomes successfully reduced the bacterial load in the urine, bladder, and kidney tissues of mice infected with MDR *E. coli*, while also lowering the levels of inflammatory cytokines and oxidative stress markers in serum and urine samples. Additionally, the nanophytosomes improved histopathological changes in bladder and kidney tissues caused by UTI without causing any toxicity or adverse effects on kidney function or hematological parameters.

**Conclusion:**

Our research introduces a cost-effective and innovative approach to addressing UTIs caused by MDR *E. coli* by the use of rosemary oil, a natural antimicrobial agent encapsulated in nanophytosomes. This strategy not only demonstrates proven therapeutic efficacy in UTI animal models but also promotes the adoption of sustainable medical approaches. CS-nanophytosomes provides a sustainable alternative therapeutic option to combat MDR UTIs.

**Graphical Abstract:**

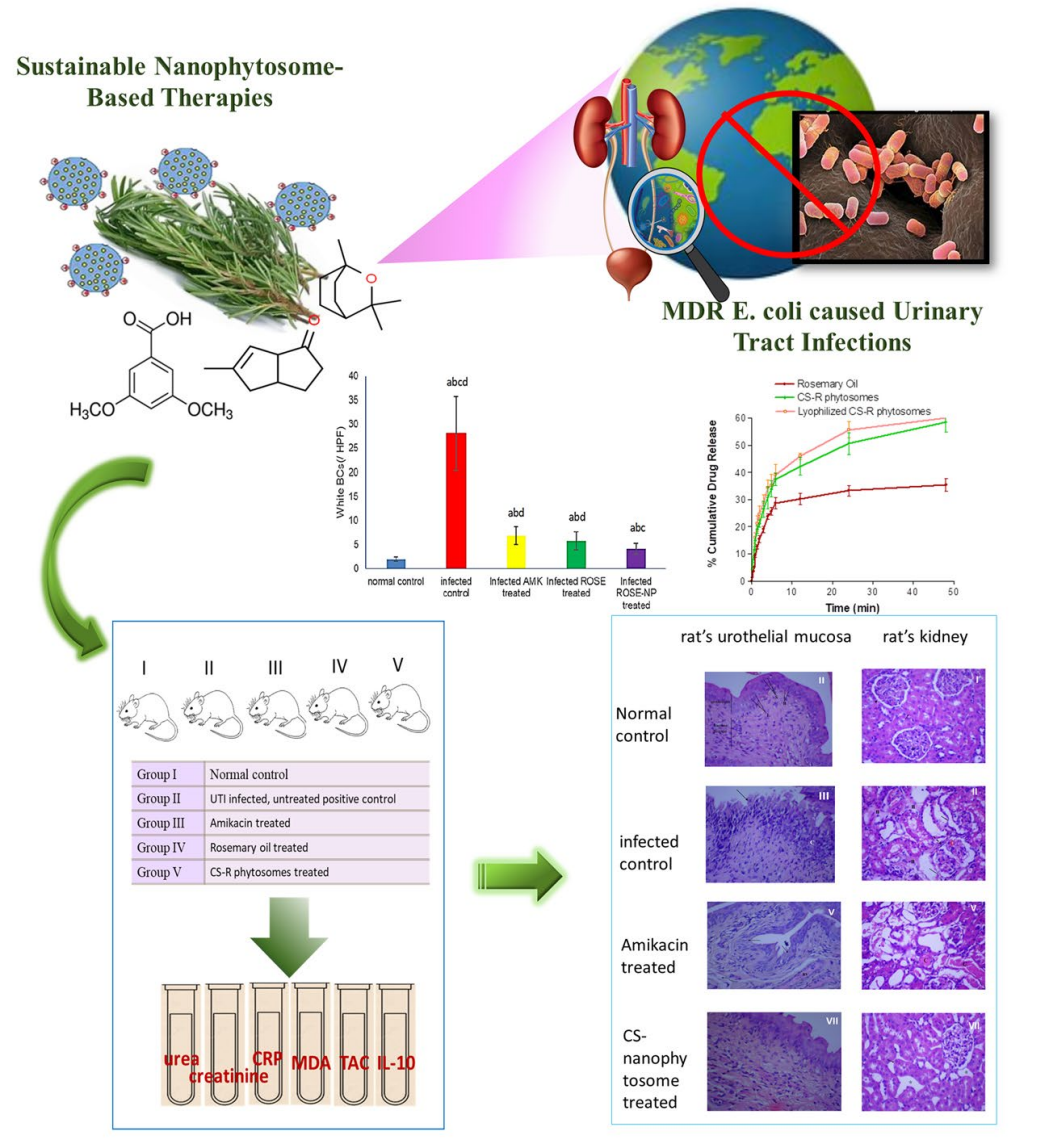

**Supplementary Information:**

The online version contains supplementary material available at 10.1186/s12951-024-03006-1.

## Background

Urinary tract infections (UTIs) are prevalent bacterial infections, affecting more than one million individuals worldwide each year. UTIs rank second among nosocomial infections, after lower respiratory tract infections in developing nations [1]. UTIs are common in both community and hospital settings, and high resistance rates to antibiotics are a major factor that influences their treatment. A study on the global incidence of UTIs showed that the prevalence of healthcare-associated UTIs is 12.9% in the U.S., 19.6% in Europe, and 24% in developing countries, with high medical costs [2]. Numerous pathogens, including both Gram-positive and Gram-negative bacteria as well as fungi, can cause UTIs. The most common pathogens infecting the urinary tract are uropathogenic *Escherichia coli* strains, which express a range of virulence factors, such as adhesins, fimbriae, biofilms, and flagella [3]. This causes resistance to the first-line antimicrobial treatment with trimethoprim-sulfamethoxazole necessitates the implementation of antimicrobial stewardship principles during treatment. Many UTI patients also experience recurrent UTIs caused by the same strain of bacteria. The economic impact of these resistant microbial infections is a significant global medical burden and challenge. As a result, finding new antibiotics or alternative regimens is a top priority for medical researchers and clinicians [4]. A growing number of the focus was on the natural phytochemicals due to their sustainable benefit to the environment and humans a number of natural product oils and extracts have been documented to be utilized in conjunction with currently available antibacterial substances to treat bacterial and fungal infections [5].

As part of the strategies to combat MDR in UTI pathogens, manipulating virulence factors rather than growth factors has proven to be effective and promising. One such approach is the use of natural products that are sourced from plants and other natural sources and are renewable and sustainable. They also tend to have fewer side effects and are often gentler on the human body than chemical medicines. Research has shown that many natural herbal remedies have remarkable anti-infectious effects due to their chemical diversity and biological properties, which differ from those of synthetic antibiotics [6]. Essential oils (EOs) extracted from nonwoody, aromatic plants contain a mixture of volatile molecules, including terpenes, terpenoids, and phenylpropanoids, which have been shown to disrupt bacterial quorum sensing, a major communication system responsible for pathogen virulence [7]. Their exceptional ability to disrupt cell membrane integrity and permeability, interfere with protein synthesis, and inhibit biofilm formation have been reported [8]. The antimicrobial mechanisms of herbal components against bacteria usually involve various approaches, including damaging the cell membrane and wall, inhibiting nucleic acid and protein synthesis, disrupting energy metabolism, inhibiting bacterial efflux pumps, and increasing intracellular osmotic pressure, as reviewed by Liang et al. [9]. For instance, tea tree essential oil nanoemulsion has been shown to increase drug accumulation in multidrug-resistant *E. coli* by disrupting the outer and inner membranes and inhibiting the AcrAB-TolC efflux pump, which is involved in membrane permeability [10]. On the other hand, the antimicrobial effect of rosemary is mainly by interacting with the cell membrane involving modification of the transit of electrons, altered fatty acid synthesis, disruptions in genetic material and nutrient transport, and causing cellular component leakage. Additional reaction also occurs with the protein components of the cell membrane, further contributing to its structural damage and loss of functionality [11]. Additionally, polycationic chitosan nanophytosomes may interact with the negatively charged surface of *E. coli*, disrupting the membrane and leading to the leakage of intracellular components, such as small ions, DNA, and RNA, which can be detected using a UV/Vis spectrophotometer due to their strong absorption at 260 nm [12]. However, it is important to note that the antibacterial activity of herbs can vary depending on factors like species, producing areas, harvest seasons, medicinal parts, extraction, separation, and purification processes.

Several studies have provided evidence that combining conventional antibiotics with EOs can synergistically inhibit the growth of MDR bacteria, such as methicillin-resistant *Staphylococcus aureus* [13], *Acinetobacter baumannii*, and *Enterococcus faecalis* [14]. However, finding a natural herbal medicine to treat, not prevent the MDR UTI is still on the serge. Various outcomes have been published, such as a 2024 meta-analysis of randomized controlled trials reported that the studied traditional Chinese herbal medicines treatment alone was not superior to antibiotics in the acute treatment phase of urinary tract infection or the follow-up period [15]. Another review indicated a clear benefit of cranberry products for the prevention of recurrent UTIs in women. However, this research primarily utilized cranberry for UTI prevention, alongside other Chinese and Korean herbal medicines [16]. Therefore, phytotherapy in the treatment of UTI is an urgent need. One of the promising herbs is rosemary.

The use of nanotechnology in medicine is becoming a global trend in drug delivery, particularly as a strategy to combat antibiotic-resistant and difficult-to-treat bacterial infections [17]. Nanoparticle drug delivery systems not only enhance the therapeutic efficacy of drugs but also target intracellular delivery to prevent the growth of MDR bacteria [18]. One of these delivery systems is nanostructured carriers, which have a high surface-to-volume ratio and are larger than individual molecules but smaller than microsystems. The therapeutic agent can be entrapped within the nano matrix or attached to the nanoparticle surface through conjugation or adsorption. These carriers can transport therapeutic agents to the target site and penetrate bacteria, providing unique bactericidal mechanisms. Antimicrobial phytochemicals delivered by nanocarriers are an efficient and effective way to deliver phytoactive compounds to fight infection [19]. On the other hand, chitosan is primarily used as a coating agent on the surface of nanoparticles to create positive charges for drug delivery. Its antibiofilm properties have been extensively researched. Studies have shown that lipopeptide carboxymethyl chitosan nanoparticles effectively inhibit and scavenge the formation of *Staphylococcus aureus* biofilms and the growth of surface-attached bacteria [20, 21].

Although there has been significant progress in the design of nanotechnology-based dosage forms over the past decade, there is still much potential to explore the use of this technology for the delivery of natural phytomedicines. Our research introduces a cost-effective and innovative approach to addressing UTIs caused by MDR *E. coli* by the use of rosemary oil, a natural antimicrobial agent encapsulated in nanophytosomes This strategy involves the use of rosemary oil nanophytosomes as a drug delivery system which offers the potential benefit of utilizing natural resources and biodiversity while providing an affordable treatment option and aligning with global sustainable development goals.

## Materials and methods

### Materials

High-grade rosemary leaves suitable for human consumption were purchased from local markets. Chitosan (84.7% degree of deacetylation, MW100,000-300,000), cineol, tripolyphosphate, thiobarbituric acid, and Tween 80 were obtained from Sigma‒Aldrich, USA. Labrafac™ lipophile WL 1349 was a gift from Gattefosse (Lyon, France). Other agents included mannitol powder (Blackburn, UK), stearic acid (El Gomhouria, Egypt), amikacin ampule (Amoun Pharmaceuticals, Egypt), gum acacia (Arabic Laboratory Equipment, Egypt), sodium dodecyl sulfate (SDS) (Thermo Fisher Scientific, Egypt), thiobarbituric acid (TBA) (Sigma‒Aldrich, St. Louis, MO, USA), and n-butanol (Arabic Laboratory Equipment Co. Cairo, Egypt). All other reagents and chemicals used were of pharmaceutical and analytical grade.

### Clinical isolates and identification of the Escherichia coli strain

A total of 70 identified clinical *E. coli* isolates were collected from patients with UTIs at Abukir Hospital, Abuhomos Hospital, and Mabaretelsafra Medical Lab. The collection of these isolates was approved by the ethical committee (Ethics Code No 318PM3. Supplementary file) at Damanhour University, with the consent of the participating patients, hospitals and labs. The research was performed following relevant guidelines/regulations, and informed consent was also obtained from all subjects and/or their legal guardian(s). A VITEK 2 microbial identification system (Bio-Merieux, l’Etoile, France) was used to identify the isolates. This rigorous process ensures the accuracy and reliability of the study’s results. All the samples were confirmed to be PCR-positive.

### Quantitative determination of biofilm formation

The biofilm formation of *E. coli* isolates was studied by a microtiter plate assay (96-well) [22]. First, cultures of the isolates were incubated at 37 °C overnight and then diluted 1:100 in fresh nutrient broth. Next, 200 µL of the bacterial suspension (0.5 on the McFarland scale) was added to each well of the plate and incubated at 37 °C to allow for the formation of bacterial biofilms. The negative controls were wells that contained nutrient broth only. Each treatment was replicated three times. After 24 h of incubation, the liquid contents of the wells were removed, followed by gentle washing three times with 250 mL of sterile phosphate-buffered saline (PBS; pH 7.4) to remove all nonadherent cells. The formed biofilm was stained with 150 µL of 2% Hucker crystal violet dye for 15 min at room temperature. Excess dye was rinsed off three times with tap water followed by distilled water, after which the mixture was air-dried by turning the microtiter plate upside down. The dye bound to the adherent cells was resolubilized with 160 µL of 95% (v/v) ethanol in each well, and the optical density (OD) of the biofilm was determined using an ELISA reader (STATFAX2100, Fisher Bioblock Scientific, France) at 630 nm. The adherence of the strains tested was classified into four categories: none adherent (OD ≤ OD_C)_, weakly adherent (ODc < OD ≤ 2×ODc), moderately adherent (2×ODc < OD ≤ 4×ODc), and strongly adherent (4×ODc < OD). The cutoff OD (OD_C_) was defined as three standard deviations above the mean OD of the negative control [23].

### Extraction of EO and gas chromatography/mass spectrometry (GC‒MS) analysis

The EO of Rosmarinus officinalis L. was freshly extracted from the fine powder of its dried leaves by the Clevenger hydrodistillation method. A volatile oil trap apparatus was used to collect the EO under optimal operating conditions, and the extracted rosemary EO was dried with anhydrous sodium sulfate, stored in the dark, sealed in vials, and kept in a refrigerator (4–8 °C) until use [24]. The extraction process was repeated twice. The chemical composition of the rosemary oil was analyzed using a trace GC instrument equipped with an ISQ mass spectrometric detector and a TG 5 MS analytical column (30 m × 0.32 mm id) (Thermo Fisher Scientific, Germany). Compounds were identified by comparing their mass spectrum patterns to the standard mass spectra available in the National Institute of Standards and Technology (NIST) Mass Spectra Database.

### Preparation of essential oil nanophytosomes

Chitosan nanophytosomes were prepared by the ionotropic gelation method. Chitosan is a natural polymer with high molecular weight and hydrophobic nature. To improve the solubility of chitosan, acetic acid is used as a solvent. The ionic interactions between positively charged chitosan molecules and the negatively charged acetate ions (CH₃COO⁻) from the acetic acid help to stabilize the chitosan solution. Two concentrations of CS solution (1% and 0.5% w/v) were prepared by dissolving 400 mg and 200 mg of CS, respectively, in 40 mL of 1% acetic acid and stirring overnight at 600 rpm and 60 °C. Tween 80 (1% w/v) was then added with continuous stirring for 30 min, followed by the gradual addition of rosemary EO to the mixture and stirring for another 10 min at 1,000 rpm to obtain a homogenous solution. Finally, tripolyphosphate (TPP), an ionic cross-linker, was slowly added to the mixture at a mass ratio of 4:1 (CS: TPP) and stirred at 600 rpm for 2 h to maximize the cross-linkage between CS and TPP until the formation of an opalescent coacervate [25].

We also prepared nanostructured lipid carrier nanoparticles (NSLCs), which are second-generation lipid nanoparticles containing both solid and liquid lipids. All the ingredients selected were obtained from natural sources or derived from natural ingredients for sustainability. These nanoparticles encapsulate greater amounts of drugs and stabilize the nanoparticles [26]. The lipid phase consisted of a mixture of Labrafac, stearic acid, cineol, and the tested EO. Both the lipid and aqueous phases were heated to 60 °C separately with gentle stirring. Then, the aqueous solution was slowly added to the lipid melt and stirred vigorously under a high-speed homogenizer (IKA T25, Digital ULTRA TURRAX^®^, Germany) at 20,000 rpm for 10 min, followed by sonication for 5 min at 60 °C. The hot NSLC dispersion was cooled to room temperature with continuous stirring at 600 rpm overnight. The compositions of these two types of nanoparticle formulations are presented in Table 1.

**Table 1:** The compositions of CS-Nanophytosomes and NSLC-Nanophytosomes

**CS-Nanophytosomes**						
	Chitosan Solution		Rosemary Oil	Tween 80		TPP
(A)	0.5% w/v 200 mg/40 mL		200 mg	800 mg		0.125% w/v 50 mg/40 mL
(B)	1% w/v 400 mg/40 mL		400 mg	800 mg		0.25% w/v 100 mg/40 mL
**NSLC-Nanophytosomes**						
	Stearic Acid	Labrafac	Cineole	Rosemary Oil	Tween 80	Water
	500 mg	300 mg	50 mg	150 mg	300 mg	8.7 mL

### Lyophilization

Freeze drying was used to preserve the homogeneity of the nanoparticles, prevent their aggregation, and increase their chemical and physical stability over extended periods [27]. The selected 0.5% CS nanophytosomes were freeze-dried using a laboratory-scale vacuum freeze dryer (HumanLab Instrument, Korea) with operating conditions of -62 °C and 0.05 mTorr. The cryoprotectant mannitol 4% (w/v), selected from the preliminary study, was added to the liquid nanoformulation before the primary freezing process in a -80 °C freezer (Thomas Scientific, US) for 24 h [28]. The frozen sample was then transferred to a freeze dryer under vacuum for 19 h to complete the lyophilization process. Deionized water (0.5 mL) was then used to reconstitute 30 mg of lyophilized nanoparticle powder and analyze the particle size [29]. Freeze-dried samples were stored in a desiccator for further analysis.

### In vitro characterization of nanoparticles

#### Particle size (PS), zeta potential (ZP), and polydispersity index (PDI)

PS, ZP, and PDI were measured using dynamic light scattering with a Zetasizer Nano ZS 90 (Malvern Instruments, UK) at room temperature and at a 90° scattering angle. To prevent multiple scattering, the samples were diluted before measurement. All measurements were performed in triplicate.

#### Entrapment efficiency (EE%)

A 500 µL sample was placed in the upper chamber of a Spin-Pure Eppendorf filter tube (MWCO 10 kDa, Sera Care, USA) and subjected to ultracentrifugation at 20,000 rpm for 45 min to separate the unentrapped oils. The filtrate was then filtered with 0.22 μm Millex filters (EMD Millipore Corporation, MA, US), and the concentration of rosemary oil was determined using a UV/VIS spectrophotometer (Shimadzu UV-1800, Japan) at λmax 258 nm.

The entrapment efficiency of rosemary oil in nanoparticles was calculated indirectly by subtracting the measured amount of unentrapped oils from the total amount of rosemary oil used in the formulation using Eq. 1.

1 $$\:\mathbf{E}\mathbf{E}\mathbf{\%}=\frac{\:\mathbf{T}\mathbf{o}\mathbf{t}\mathbf{a}\mathbf{l}\:\mathbf{a}\mathbf{m}\mathbf{o}\mathbf{u}\mathbf{n}\mathbf{t}\:\mathbf{o}\mathbf{f}\:\mathbf{E}\mathbf{O}-\mathbf{u}\mathbf{n}\mathbf{e}\mathbf{n}\mathbf{t}\mathbf{r}\mathbf{a}\mathbf{p}\mathbf{p}\mathbf{e}\mathbf{d}\:\mathbf{E}\mathbf{O}\:}{\mathbf{T}\mathbf{o}\mathbf{t}\mathbf{a}\mathbf{l}\:\mathbf{a}\mathbf{m}\mathbf{o}\mathbf{u}\mathbf{n}\mathbf{t}\:\mathbf{o}\mathbf{f}\:\mathbf{E}\mathbf{O}}$$

#### Transmission electron microscopy (TEM)

The shape and size of nanoparticles in a formulation can greatly affect their physical, chemical, and functional characteristics. To investigate these properties, the samples were examined using TEM (JEOL JEM 1230, Japan). The samples were first placed on copper grids, dried at room temperature, and then stained with a 1% tungstophosphoric acid solution before analysis. The electrical conductivity was measured using a DDS-11 A digital conductivity meter attached to a platinum electrode (Shanghai Leida Instrument Co., Shanghai, China) with an acceleration voltage of 200 kV. The average size of the nanoparticles was calculated using ImageJ software.

#### Fourier transform infrared (FT-IR) spectroscopy

The interaction between the excipients of the CS nanophytosomes and their functional groups was assessed using FT-IR. The FT-IR spectra of rosemary oil, TPP, 0.5% CS, and 0.5% CS nanophytosomes were analyzed in the range of 4000 to 450 cm^− 1^ at ambient temperature using a Cary 630 FTIR Spectrometer (Agilent, Santa Clara, CA). No further preparation was needed, as all the samples were analyzed in their original forms.

### Minimum inhibitory concentration (MIC) and biofilm inhibition

To determine the MICs of plain rosemary oil, nanophytosomes, and negative controls against *E. coli*, samples with a concentration of 2.5 mg/ml were serially diluted 2-fold with sterile nutrient broth, and then 200 µL was transferred into the wells of a microtiter plate. Uninoculated wells containing plain rosemary oil or nanophytosomes were used as negative controls [30]. The MIC was determined as the lowest concentration that inhibited the visible growth of *E. coli* on the plates.

To evaluate the biofilm inhibitory effect of rosemary oil and its nanoformulations on strong biofilms, the microtiter plate method was used to assess ¾, ½, and ¼ MIC of the formulations. Different concentrations of the formulations were prepared in 100 µL of tryptic soy broth (TSB) and subsequently added to wells containing 100 µL of the inoculum. After incubating for 24 h at 37 °C, the biofilms were quantified using the same method as previously reported [31]. An untreated bacterial suspension was used as a control. Each experiment was conducted in triplicate.

### In vitro drug release study

Modified Franz diffusion cells (Nanseng Lab Glass, Taipei, Taiwan) were used to study the drug release of the selected CS nanophytosomes and their freeze-dried products compared to that of plain rosemary oil [32]. The receptor chamber of the diffusion cells was enclosed by a glass jacket, allowing water at 37 °C to flow through a circulating thermostatic water bath (Thermo Scientific, US) and maintain a constant temperature in the receptor medium. The receptor compartment had a volume of 21 mL and was filled with 70% prewarmed phosphate buffer (pH 7.4) and 30% methanol to achieve “sink” conditions and ensure that the drug concentration does not reach the solubility limit in the receptor compartment throughout the experiment. The amount of methanol required was calculated by a preliminary experiment of rosemary oil solubility in the release medium and methanol. A dialysis membrane with a molecular weight cutoff of 12,000–14,000 (Spectra/Pro^®^ Spectrum Laboratories, Inc., California, US) was presoaked in deionized water overnight, washed several times, and then allowed to equilibrate between the donor and receptor compartments with the receptor medium for 30 min before the donor samples were introduced. The donor compartment contained 0.5 mL of the tested samples at a concentration of 3 mg/mL. Plain rosemary oil was dispensed in a receptor medium, while the freeze-dried CS nanophytosomes were freshly reconstituted immediately before the experiment. All diffusion cells were placed on a multicentered magnetic plate (AlNasr instrument, Egypt) with the receptor medium constantly stirred at 100 rpm.

At predetermined time intervals, 0.5 mL of the receptor medium was removed after the stirrer was stopped, and the mixture was immediately replenished with an equal volume of prewarmed, fresh medium to maintain a constant volume before the initiation of stirring. The experiment was conducted for 48 h. All the released samples were filtered and diluted. The in vitro release study of rosemary oil faces challenges due to the diverse and complex composition of essential oils. To address this, targeted selection of the major constituents was used for the in vitro release study. The GC–MS analysis and mass spectra of the NIST database indicated a high concentration of 3,5-dimethoxybenzoic acid (96.56%) in the extracted rosemary oil. Therefore, a diluted rosemary oil sample with methanol was scanned using a UV spectrophotometer over the wavelength range of 200–400 nm. The absorption maximum (λmax) was identified at 258 nm, and a calibration curve was constructed accordingly. The experiment was replicated 6 times. We also analyzed the in vitro release mechanism of the zero-order, first-order, Higuchi Korsmeyer–Peppa, and Hixson–Crowell models based on the correlation coefficient (R^2^). The model that best fits the data was selected based on the highest R^2^.

### Physical stability study

Physical stability studies were performed on the selected 0.5% CS nanophytosomes and their freeze-dried products at room temperature for three months under normal shelf conditions. All the samples were stored in closed USP-type I glass containers. The changes in the PS, ZP, and PDI of the stored formulations were examined monthly. The presence of phase separation and turbidity was also visually inspected. Stored freeze-dried samples were redispersed with deionized water just before being assayed.

### In vivo urinary tract infection (UTI) study

### Experimental animals

Forty female Wistar rats weighing 150–200 g were included in the study. Female rats were selected to enhance the precision of simulating the major prevalence of sex in human UTIs. The animals were housed in well-ventilated rooms inside polypropylene animal cages with wood shavings under standard environmental conditions of 22–25 °C and a 12-h light/dark cycle. The animals had free access to water and a standard chow diet. All animal handling procedures and care complied with the ARRIVE guidelines and were carried out according to the Guide for the Care and Use of Laboratory Animals [33].

After one week of acclimatization, the rats were randomly divided into five groups. Group I was the normal control group, in which the rats were fed and given water normally. The remaining 32 rats were subjected to urinary tract infection (UTI) induction. These rats were anesthetized with 3% isoflurane first and then injected with 50 µL of bacterial suspension containing 1 × 10^8^ colony-forming units (CFUs) of viable *E. coli* into the urethra directly to minimize complications [34]. These infected rats were further assigned to four groups. Group II was an untreated positive control group that was divided into two subgroups: Group IIa served as a positive control for intraperitoneal (i.p.)-treated rats and received 0.5 mL of 0.9% saline i.p. daily, while Group IIb served as a positive control for orally treated rats and received 1 mL of oral 2% gum acacia daily. Group III was treated with amikacin i.p. at a high dose of 60 mg/kg/day [35], and Group IV was treated with rosemary oil orally at a dose of 1 mL (10 mg/kg)/day. Group V was treated orally with 0.5% CS nanophytosomes at a dose of 1 mL (equivalent to 10 mg/kg conventional rosemary oil)/day [36]. The doses of rosemary oil and its nanoparticles were suspended in 2% gum acacia. All the treatments were administered once daily for seven consecutive days.

### Biochemical analysis of urine and serum samples

The rats were placed in metabolic cages and kept separate to collect urine samples for 24 h after the last dose. The rats were then weighed and anesthetized with thiopental sodium (30 mg/kg, i.p.). Blood samples were collected from the abdominal aortae and allowed to coagulate for 1 h at 37 °C until clot retraction occurred. The sera were separated by centrifugation at 3000 rpm for 15 min and divided into portions to measure the levels of creatinine, urea, and C-reactive protein (CRP).

Following blood collection of the last sample, both the kidneys and the urinary bladder were immediately removed and weighed. The right kidney and urinary bladder were fixed in 10% formalin-saline for histopathological examination. The left kidney from each rat was washed with ice-cold isotonic saline, homogenized in phosphate-buffered saline (0.1 M PBS, pH 7.4) to obtain 10% homogenates, divided into portions, and stored at -80 °C until use in biochemical assays; these samples included malondialdehyde (MDA), total antioxidant capacity (TAC), and interleukin-10 (IL-10) levels.

### Assays for determining the serum creatinine, urea, and CRP levels

Serum creatinine was measured using a spectrophotometric assay based on the Buffered Kinetic Jaffé reaction without deproteinization, which involves the reaction of creatinine with picric acid in an alkaline environment to form a yellow‒red complex [37]. For urea, we used the urease colorimetric method, where urea is hydrolyzed in the presence of urease and water into CO_2_ and ammonia, which then produces a colored complex at an alkaline pH. Serum CRP levels were determined using a quantitative sandwich ELISA kit (Cat. No. RH951CRP01R; Biovendor Research & Diagnostic Products, Czech Republic) [38]. The color intensity was measured at 450 nm against a blank using an ELISA reader (Tecan, Infinite 200 PRO, Switzerland).

### Assays of MDA, TAC, and IL-10

***The*** MDA level in renal tissue homogenate was measured using a colorimetric assay based on the reaction between MDA and thiobarbituric acid [39]. The level of TAC was determined using a spectrophotometric assay based on the reaction of antioxidants in the sample with hydrogen peroxide (H_2_O_2_), and the residual TAC was determined by an enzymatic reaction that converts 3,5 dichloro-2-hydroxy benzene sulfonates into a colored product measurable at 505 nm [40]. The level of IL-10 was measured using a quantitative sandwich ELISA kit (Cat No. MBS8244601, My BioSource Co., California, USA) [41]. The optical density (OD) of the samples was measured at 450 nm using a microplate ELISA reader (Tecan, Infinite 200 PRO, Switzerland).

### Histopathological examination

The right kidney and urinary bladder specimens from all the rat groups were removed and preserved in 10% neutral formalin for a minimum of three days before being processed and embedded in paraffin. Sections with a thickness of 5 μm were cut, stained with hematoxylin and eosin (H&E), and examined under a microscope. Histopathological features such as inflammation, fibrosis, cellular changes, and tissue architecture are assessed. These standardized assessments are consistently applied across various treatment groups for a comprehensive comparison.

### Statistical analysis

All the data are presented as the mean ± standard deviation (SD). Statistical analysis was performed using Student’s t-test or one-way ANOVA followed by a post hoc test, as appropriate, using GraphPad Prism version 8.0.0 for Windows (GraphPad Software, San Diego, California, USA). Some data are reported as percentages. For all the statistical tests, differences were considered significant at *p* < 0.0 1 and highly significant at *p* < 0.001, with 95% confidence intervals. Microsoft Excel and GraphPad Prism were used to create the artwork.

## Results and discussion

### Identification of clinical isolates and biofilm formation

Biofilm formation is a microbial survival strategy, and these surface-associated microbial cells facilitate the adherence of microorganisms to living and inanimate surfaces. In the human host, biofilms can serve as a reservoir for spreading new infections and increasing bacterial resistance to antibiotics. Among the *E. coli* isolates collected in this study, 28.6%, 32.9%, 24.3%, and 14.3% had strong, moderate, weak, and no biofilm formers, respectively. These data were in agreement with those of another study of biofilm formation by uropathogenic *E. coli*, which showed 23.6% highly positive, 26.3% moderately positive, and 50% weakly positive biofilm formation in the 100 tested strains [42]. The virulence of these uropathogenic *E. coli* fimbrial adhesins was reported to be triggered by Ag43, which might be the major contributing factor to long-term persistence after the establishment of an initial infection, and the Ag43a gene was responsible for a strong aggregation phenotype that promoted significant biofilm growth [43].

**Fig. 1:**
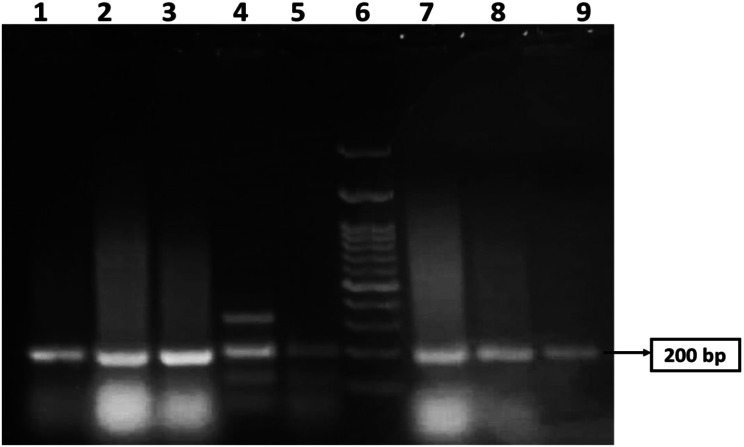
PCR amplification of csgA gene with a size 200 bp in MDR *E.coli* revealed by agarose gel analysis. Lane 6 was loaded with a 200 bp DNA ladder. Samples were loaded in lanes 1, 2, 3, 4, 5, 7, 8, and 9. All lanes are positive for the csgA gene. The original image is presented in Supplementary Fig. 1

Figure 1 shows the 200 bp PCR-amplified csgA gene in *E. coli*, which was visualized via gel electrophoresis analysis. The samples were loaded in lanes 1, 2, 3, 4, 5, 7, 8, and 9, while lane 6 was loaded with a 200 bp DNA ladder. All the isolates were positive for the csgA gene. An image-editing tool (www.irfanview.net) was used to enhance the representation of the image (original image, supplementary file).

### GC‒MS analysis of rosemary oil

Phytochemical constituents identified in dried leaf extracts of *Rosmarinus officinalis L.* by GC‒MS analysis showed that high concentrations in the extracted rosemary oil were 3,5-dimethoxybenzoic acid (96.56%), eucalyptol (25.68%), and bicyclo[3.3.0]oct-2-en-6-one, 3-methyl (17.84%) (Table 2). This finding suggests that rosemary oil contains a significant amount of oxygenated compounds and phenylpropanoids, which are known for their potential therapeutic properties. Oxygenated compounds and phenylpropanoids have been reported to exhibit various biological activities, such as antioxidant, antimicrobial, and anti-inflammatory effects [44]. GC-MS chromatogram of rosemary oil is available in supplementary file.

**Table 2:**
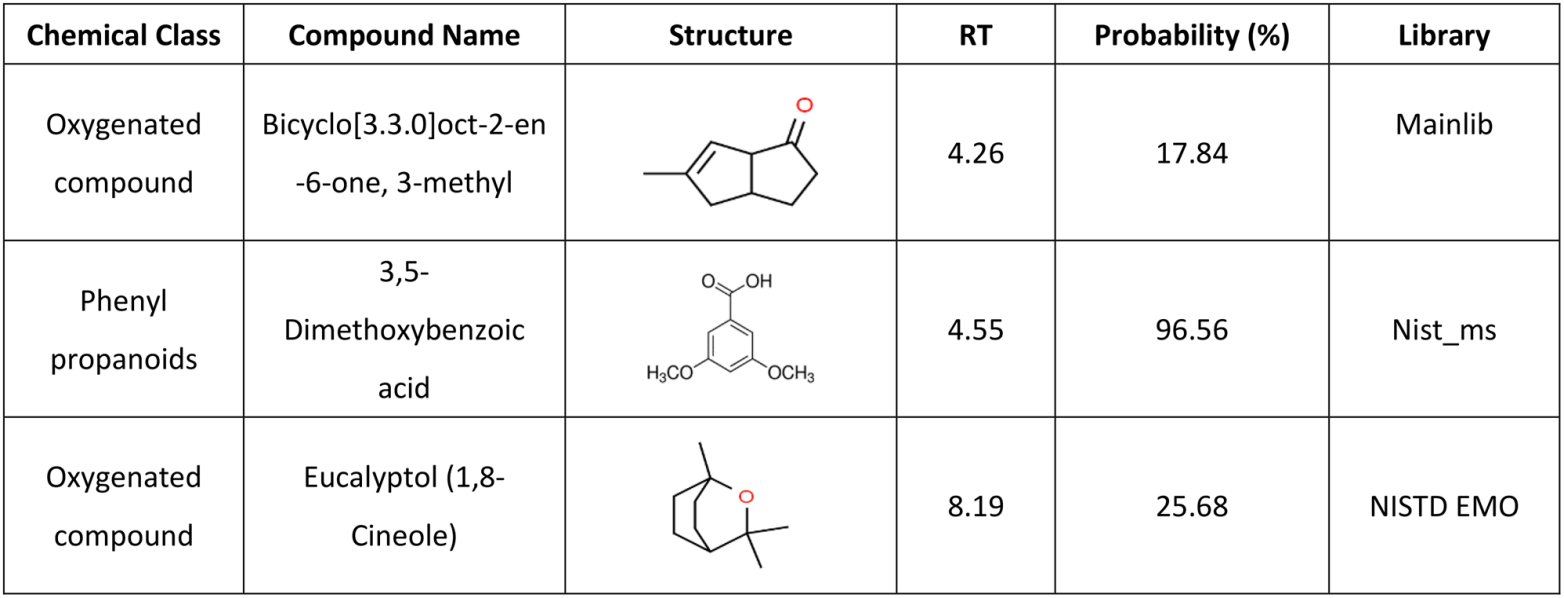
Phytochemical constituents identified in dried leaf extracts of *Rosmarinus officinalis L.* by GC–MS analysis and mass spectra of the NIST database

### Transmission electron microscopy (TEM)

**Fig. 2:**
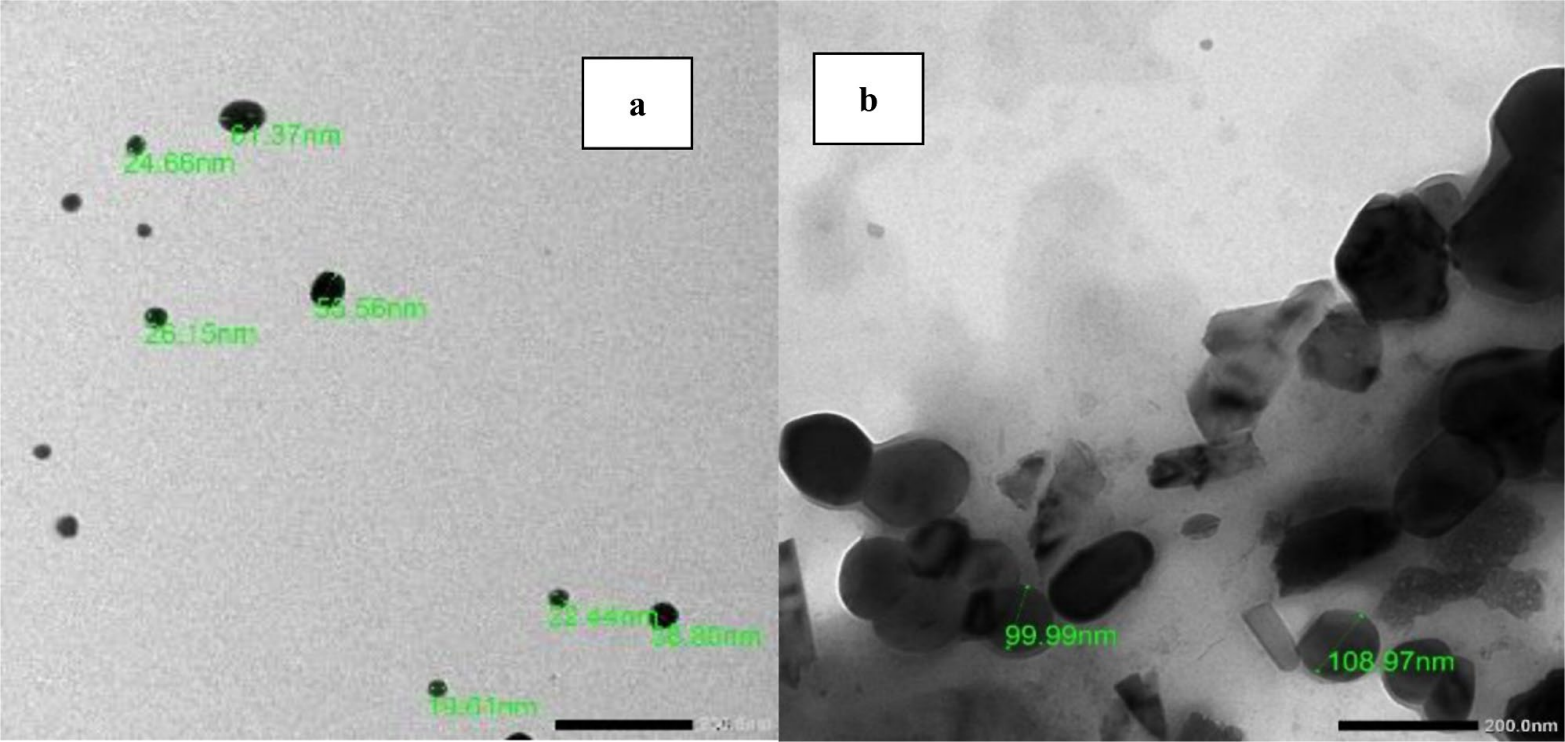
TEM images of (**A**) 0.5% CS-Nanophytosomes, and (**B**) NSLC-Nanophytosomes

Figure 2 shows TEM images of freshly prepared (a) 0.5% CS nanophytosomes and (b) NSLC nanophytosomes. The CS nanophytosomes had a well-defined spherical shape with an average size of approximately 26 nm and were evenly distributed without any agglomeration. In contrast, the NSLC nanophytosomes were larger and less uniform in shape. The particle size and shape results of the nanoparticles are consistent with the findings of our previous work [45].

### Particle size (PS), zeta potential (ZP), polydispersity index (PDI), and entrapment efficiency (EE%)

Table 3 shows that the NSLC nanophytosomes had a significantly greater PS (176.70 ± 12.30 nm) than did the 0.5% CS and 1% CS nanophytosomes, which had PSs of 22.54 ± 2.92 nm and 34.59 ± 4.00 nm, respectively (one-way ANOVA, *p* < 0.001). This difference may be due to the composition of NSLC nanophytosomes, which includes a mixture of solid and liquid lipids. In contrast, the CS nanophytosomes are primarily composed of CS. The PS significantly increased when the CS concentration was increased from 0.5 to 1% (t-test, *p* < 0.001).

**Table 3:** Comparison of NSLC-Nanophytosomes and CS-Nanophytosomes in terms of particle size (PS), zeta potential (ZP), polydispersity distribution index (PDI), and entrapment efficiency (EE%). (*n* = 3)

Nanophytosomes	PS (nm)	ZP (mV)	PDI	EE%
NSLC	176.70 ± 12.30	-32.71 ± 2.07	0.45 ± 0.01	93.47 ± 0.90%
0.5% CS	22.54 ± 2.92	11.5 ± 0.05	0.57 ± 0.14	93.12 ± 1.05%
1% CS	34.59 ± 4.00	12.57 ± 0.01	0.69 ± 0.23	95.51 ± 026%

The surface charge of nanoparticles in colloids can be used to predict their long-term stability. The NSLC nanophytosomes exhibited a negative ZP of -32.71 ± 2.07 mV, while the 0.5% CS nanophytosomes had a positive ZP of 11.5 ± 0.05 mV due to the unbranched cationic nature of the CS. The stronger surface charges of NSLC nanophytosomes suggest that they may be more stable, as the nanoparticles can repel each other more effectively. In contrast, the weaker surface charges around the CS nanophytosomes may not be sufficient to prevent aggregation over time, suggesting the necessity of lyophilization for long-term storage stability.

Compared with 0.5% CS nanophytosomes, the NSLC nanophytosomes had significantly lower PDI values (0.45 ± 0.01 and 0.57 ± 0.14, respectively) (one-way ANOVA, *p* < 0.001). This means that the NSLC nanophytosomes were more monodisperse than the CS nanophytosomes were. Moreover, increasing the CS concentration resulted in a more heterogeneous mixture, as indicated by a significant increase in the PDI (t-test, *p* < 0.05). In addition, both the NSLC and CS nanophytosomes exhibited high entrapment efficiencies of 93.47 ± 0.90% and 93.12 ± 1.05%, respectively. However, due to the potential benefits of smaller particle sizes in increasing drug loading, 0.5% CS nanophytosomes were selected for further investigation.

### Lyophilization of nanoparticles

Freeze-drying is a technique that converts colloidal dispersions into dry powders with enhanced long-term storage stability. The process involves freezing the nanoformulation, reducing the pressure, and removing water by sublimation and desorption under vacuum. However, freeze-drying process poses the challenge of maintaining the original formulation structure, as ice nucleation during the process can alter the formulation morphology, cause physical collapse, and induce colloidal instability and nanoparticle aggregation [46]. To overcome these problems, a cryoprotectant, such as mannitol, can be used to immobilize nanoparticles in an amorphous matrix by replacing water molecules and preventing sample collapse due to osmotic pressure and stress. The lyophilization process has yielded soft, loose flake appearance, free-flowing CS nanophytosomes powder with short resuspension time. The reduced moisture content through lyophilization enhances stability and ensures a longer shelf life by preventing microbial growth and chemical degradation. Upon reconstitution, the lyophilized powder quickly disperses, forming a homogenous solution, which is crucial for efficient drug delivery and patient compliance.

### Minimum inhibitory concentration (MIC) and biofilm inhibition

Table 4 presents the results of MIC testing of CS and NSLC nanophytosomes against *E. coli* compared to those of rosemary oil. The data showed that both types of nanophytosomes had significantly lower MICs against *E. coli* than rosemary oil alone, with a reduction rate of 45.8% for NSLC nanophytosomes and 87.5% for 0.5% CS nanophytosomes. Further Mann‒Whitney tests confirmed that the MIC was significant lower for CS phytosomes than for NSLC phytosomes (*p* < 0.01), which may be attributed to the synergistic effect of CS and rosemary oil when combined in nanophytosomes. Consequently, CS nanophytosomes were selected for biofilm inhibition testing.

**Table 4:** MICs of pure rosemary oil, NSLC-Nanophytosome and CS-Nanophytosomes against the tested *E. coli* pathogens. (*n* = 3)

MIC (µg/ml)	Rosemary oil	NSLC-Nanophytosomes	CS-Nanophytosomes
Min. – Max	312.50–625.0	312.50–312.50	78.0–78.0
Mean ± SD	576.92 ± 117.35	312.50 ± 0.0	78.0 ± 0.0
**% MIC Reduction**		↓45.8%	↓87.5%

The outer membrane of *E. coli* has a unique structure that acts as a selective barrier, combining a highly hydrophobic lipid bilayer and pore-forming proteins with specific size exclusion properties. Therefore, nanopartilces can enter the outer membrane through either a lipid-mediated route or general diffusion porins for hydrophilic entry [47]. The small particles of CS nanophytosomes are able to utilize the porin-mediated permeability pathway to gain access to the cells of *E. coli* and interact with the water-soluble proteins and nucleic acids within the bacterial cell, resulting in a significantly reduced MIC against *E. coli*. In contrast, larger particles of NSLC nanophytosomes have to diffuse across the lipid bilayer of the outer membrane, a more challenging and rate-limiting process that contributes to the observed variation in the antimicrobial efficacy between the two types of nanophytosomes.

**Fig. 3:**
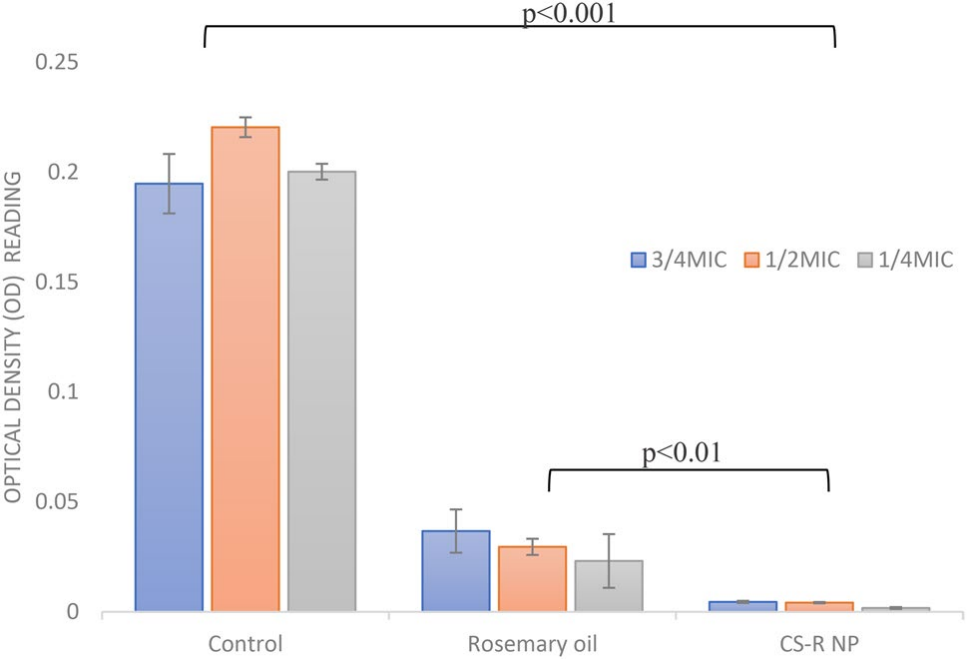
Biofilm inhibition of the rosemary oil and 0.5% CS-Nanophytosomes on strong biofilm forming MDR *E. coli*. (*n* = 3)

To evaluate the efficacy of sub-MICs of rosemary oil and 0.5% CS phytosomes at ¾ MIC, ½ MIC, and ¼ MIC in inhibiting the formation of strong biofilms produced by *E. coli*, a crystal violet staining assay was used. Compared with the control, the CS nanophytosomes significantly inhibited biofilm formation at all concentrations (one-way ANOVA, *p* < 0.001), as shown in Fig. 3. Compared with those of rosemary oil, the biofilm formation of the CS nanophytosomes also substantially decreased at all concentrations (*p* < 0.01). The significant differences observed between ¼ MIC and ½ MIC, as well as between ¼ MIC and ¾ MIC (*p* < 0.001), of the CS nanophytosomes suggested that lower sub-MIC concentrations of CS phytosomes may be more effective at inhibiting biofilms produced by MDR *E. coli*. However, no significant difference was found between the ½ MIC and ¾ MIC (*p* > 0.05), indicating that increasing the concentration of CS nanophytosomes beyond a certain point may not provide any additional benefits in terms of biofilm inhibition. Overall, these results suggest that the use of sub-MIC concentrations of CS nanophytosomes could be a promising strategy for preventing the formation of strong biofilms by MDR *E. coli*. Furthermore, the addition of chitosan into nanophytosomes enhances its ability to penetrate the biofilm matrix more effectively through electrostatic interactions between positively charged chitosan molecules and negatively charged biofilm constituents which contain extracellular polysaccharides, proteins, and DNA [48]. On the other hand, rosemary oil contains bioactive metabolites with antimicrobial properties that could interact with bacterial cell membranes, disrupt genetic material and nutrient transport leading to compromised bacterial cell functionality and structural integrity [49]. Therefore, incorporating rosemary oil into nanoformulation may augment synergistic antimicrobial efficacy.

### Fourier transform infrared (FT-IR) spectroscopy

**Fig. 4:**
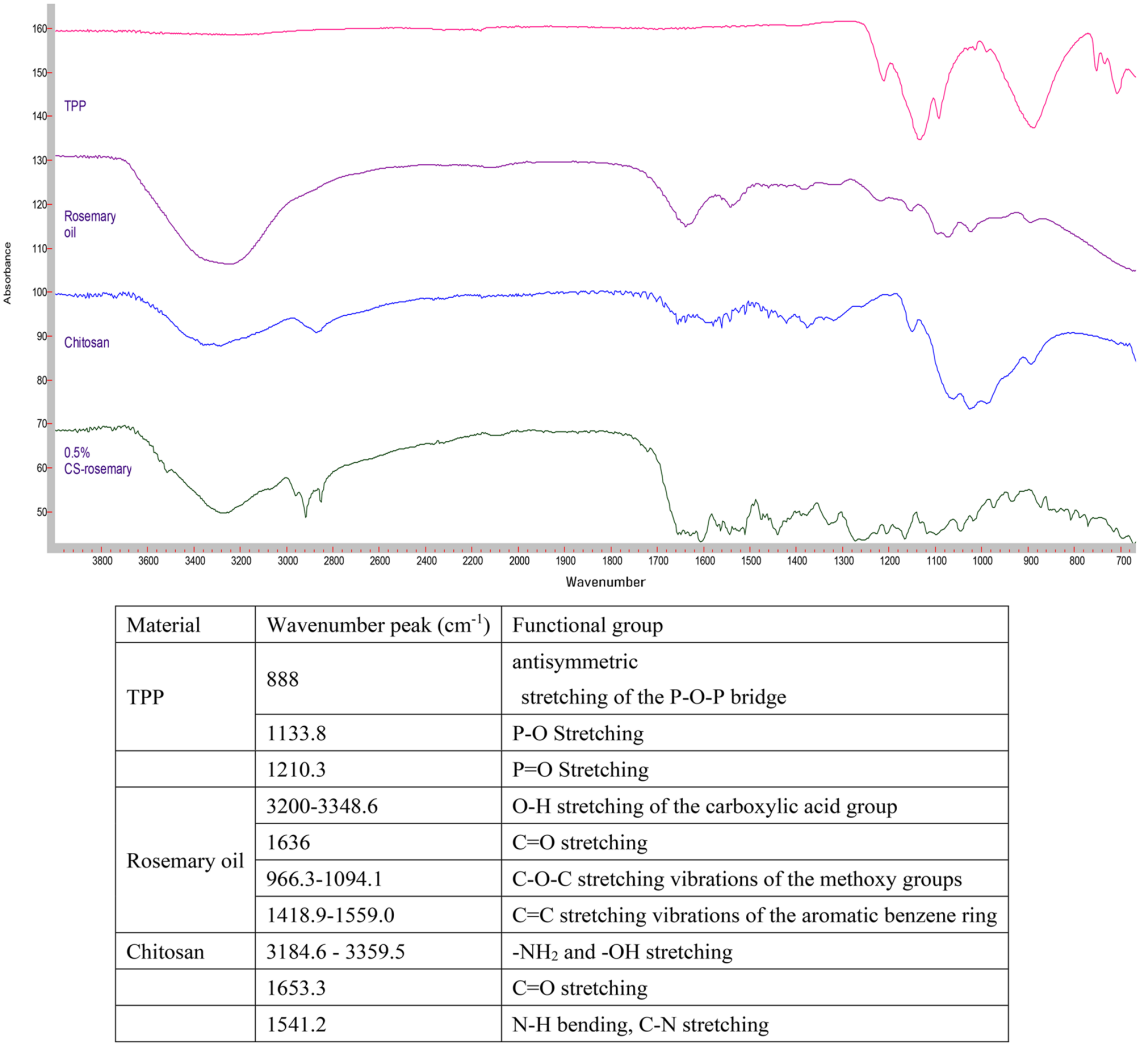
FTIR spectra of TPP, rosemary oil, CS, and 0.5% CS-Nanophytosomes

The FTIR spectra of TPP, rosemary oil, CS, and 0.5% CS nanophytosomes are presented in Fig. 4. The phosphate groups in the TPP molecule exhibit peaks at 888 cm^− 1^, 1133.8 cm^− 1^, 707.3 cm^− 1^, 752.4 cm^− 1^, and 1210.3 cm^− 1^ due to their different vibrational modes [50]. The FTIR spectrum of the extracted rosemary oil showed the characteristic absorption peaks of 3,5-Dimethoxybenzoic acid which was the major component of the extracted rosemary oil. It is an aromatic carboxylic acid with two methoxy (OCH3) substituents at the 3 and 5 positions of the benzene ring [51]. A broad absorption band observed at around 3200–3348.6 cm^− 1^ corresponding to the O-H stretching vibration of the carboxylic acid group. A strong absorption peak at 1636 cm^− 1^ corresponding to carbonyl double bond (C = O) stretching. Many absorption peaks appeared around 966.3–1094.1 cm^− 1^ were related to the C-O-C stretching vibrations of the methoxy groups. There were also multiple absorption bands in between 1418.9 and 1559.0 cm^− 1^ corresponding to the C = C stretching vibrations of the aromatic benzene ring. Our FTIR study result was similar to the published literature on rosemary oil [52]. FTIR absorption peaks in rosemary oil is available in the supplementary files.

On the other hand, the spectrum of pure chitosan exhibited the characteristic broadband with peaks around 3184.6–3359.5 cm^− 1^ due to amino (-NH_2_) and hydroxyl (-OH) stretching. A weak band with a peak at 1653.3 cm^− 1^ corresponds to the C = O stretching vibration and a strong band at 1541.2 cm^− 1^ indicates the presence of an amide bond with the N-H bending vibration and C-N stretching vibration of the amide groups [53]. Comparing the FTIR spectrum of the pure chitosan and CS-nanophytosomes, we identified slightly changes in the absorption bands, their positions, intensities, and shapes which can provide insights into the nature and extent of the ionic interactions occurring in the CS-nanophytosomes. These changes were demonstrated by the shifts in several characteristic absorption bands in the FTIR spectrum. The shape of the absorption bands of CS-nanophytosomes were becoming broader and more complex compared to the pure chitosan due to the presence of multiple overlapping vibrations. In addition, a new absorption band appeared at 1517.7 cm^− 1^ indicated the formation of ionic interactions between the amino group of chitosan and the carboxylate group of rosemary oil, corresponding to the asymmetric stretching of the carboxylate group.

### In vitro drug release

**Fig. 5:**
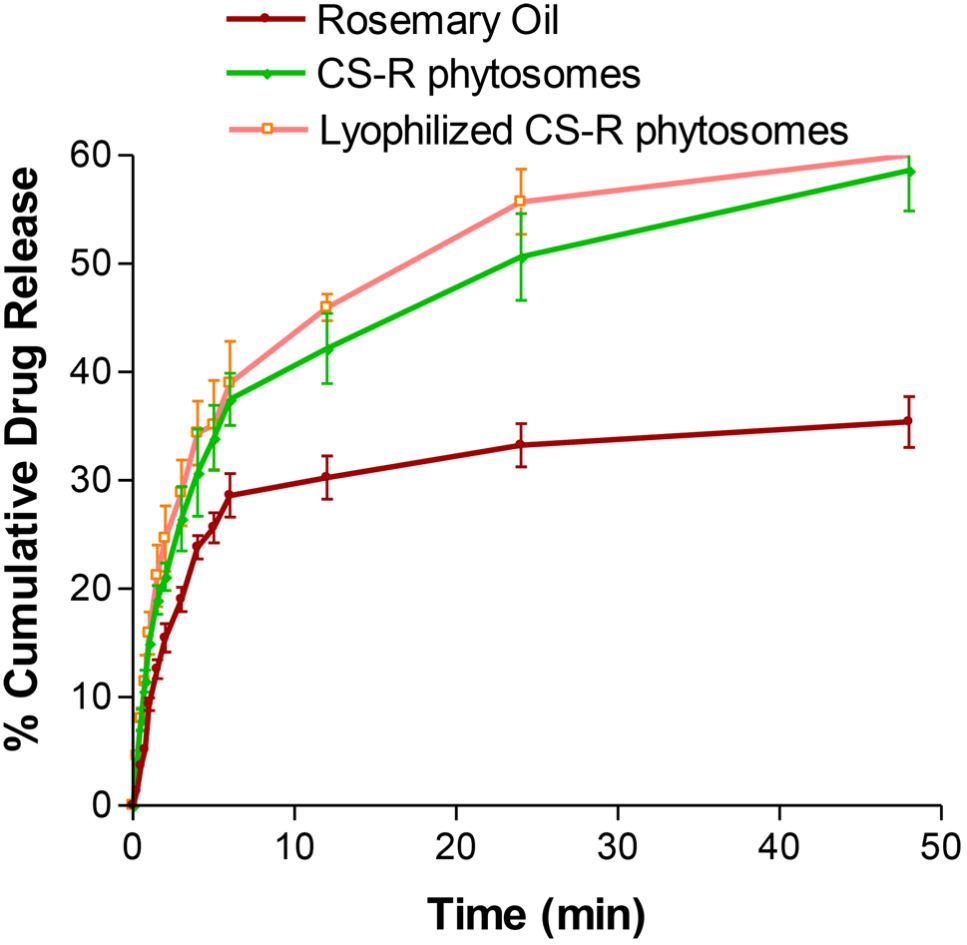
Comparison of in vitro drug release profiles between rosemary oil, CS-Nanophytosomes, and lyophilized CS-Nanophytosomes over a 48-h period (*n* = 6)

The release of rosemary oil, CS nanophytosomes, and lyophilized CS nanophytosomes over a 48-h period is shown in Fig. 5. Pure rosemary oil had a significantly lower percentage of 35.4 ± 2.36% release over 48 h, while the CS nanophytosomes and lyophilized CS nanophytosomes had markedly greater percentages of 58.6 ± 3.69% and 56.9 ± 5.01%, respectively (*p* < 0.001). This can be attributed to the hydrophilic nature of the nanophytosomes because the oil is trapped in the chitosan nanoparticles; therefore, an increase in the release of rosemary oil from the nanophytosomes occurs when they are in contact with water. Furthermore, there was no statistically significant difference between the drug release of the unlyophilized and reconstituted lyophilized CS nanophytosomes (t-test, *p* > 0.05). These results suggest that the freeze-drying process did not adversely affect drug release or induce any significant changes in the nanophytosomes.

Worth noting that the drug release profiles of all the tested samples showed an initial rapid release within the first 6 h, followed by a more sustained release. This initial burst release of the drug could promptly increase the desired plasma concentration in a short time, which is crucial for effectively combating infections. Moreover, the subsequent sustained release after 6 h might provide additional benefits by maintaining the dose for a longer time, thereby reducing the potential for side effects. These findings also highlight the potential of CS nanophytosomes as a promising drug delivery system.

The drug release kinetics of the CS nanophytosomes were analyzed using five mathematical models: zero order, first order, Higuchi, Korsmeyer–Peppas, and Hixson–Crowell [54]. The model with the highest correlation coefficient (R^2^) was considered the best-fitting model. The result showed that the overall drug release of the CS nanophytosomes over 48 h was best fitted by the first-order kinetic model, with an R^2^ of 0. 0.9807, indicating that the rate of drug release is directly proportional to the amount of drug remaining in the formulation. However, the drug release exhibited two patterns, with an initial fast release followed by a later steady release. Therefore, the kinetic models were reapplied to these two patterns separately. For both the initial rapid release within 6 h and the subsequent slow release after up to 48 h, the Higuchi model was the best fit, with R^2^ values of 0.9915 and 0.9938, respectively. This suggest that drug release from CS nanophytosomes is strongly influenced by diffusion through the chitosan hydrogel matrix in the nanoformulation.

### Physical stability study

The effects of lyophilization on the stability of the CS nanophytosomes were evaluated by measuring the PS, ZP, and PDI for 3 months at room temperature (Table 5). The data showed that there was no significant difference in the PS, ZP, or PDI between unlyophilized and lyophilized CS nanophytosomes (*p* > 0.05). However, upon storage, both samples exhibited significant changes in the PS and ZP (*p* < 0.001). The PS increased from 22.54 ± 2.92 nm to 339.33 ± 23.16 nm for unlyophilized CS nanophytosomes and from 45.66 ± 3.26 nm to 352 ± 30.25 nm for lyophilized CS-R nanophytosomes in three months. Although there were no visible physical changes in any of the samples stored for 3 months, it is expected that reorganization of inter/intramolecular hydrogen bonding and additional intermolecular entanglement of the CS particles might have occurred during long-term storage, leading to particle agglomeration. Additionally, the residual moisture in the freeze-dried samples could also affect the PS of the CS nanophytosomes. The ZP of the lyophilized CS nanophytosomes was reduced from a fresh sample of 12.36 ± 0.14 to 6.03 ± 0.05 mV after three months. The presence of more neutral surface charges around the CS nanophytosomes during storage might not be able to prevent aggregation due to the absence of an interparticle repulsive force. Furthermore, a narrowing of the PDI was observed in both unlyophilized and lyophilized CS nanophytosomes after three months, which might be attributed to the presence of more unified larger nanoparticles.

**Table 5:** Three-month stability analysis of particle size (PS), zeta potential (ZP), and polydispersity index (PDI) between freshly prepared and lyophilized CS-Nanophytosomes. (*n* = 6)

Time	Nanophytosomes	PS (nm)	ZP (mV)	PDI
Fresh sample	Unlyophilized	22.54 ± 2.92	11.5 ± 0.05	0.57 ± 0.14
	Lyophilized	45.66 ± 3.26	12.36 ± 0.14	0.53 ± 0.21
1-month storage	Unlyophilized	30.69 ± 3.07	9.32 ± 0.06	0.56 ± 0.11
	Lyophilized	59.45 ± 11.36	11.81 ± 0.21	0.46 ± 0.06
2-month storage	Unlyophilized	208.6 ± 37.62	8.62 ± 1.10	0.59 ± 0.09
	Lyophilized	296 ± 24.01	6.78 ± 0.51	0.43 ± 0.04
3-month storage	Unlyophilized	339.33 ± 23.16	8.03 ± 0.05	0.44 ± 0.03
	Lyophilized	352 ± 30.25	6.03 ± 0.05	0.23 ± 0.01

In general, nanoparticles have a high surface-to-volume ratio, which makes them prone to aggregation over time. As the nanoparticles aggregate, the overall surface area-to-volume ratio changes, resulting in the reduction of zeta potential. During storage, ions or other molecules in the formulation may be adsorbed onto the surface of CS nanophytosomes, leading to a change in the zeta potential. The alter in PS and ZP during storage may be also due to other factors such as temperature variations, exposure to light, humidity and water content in the formulation, impacting the efficacy of rosemary nanophytosomes. To maintain a consistent zeta potential, several strategies can be used in the future studies, such as incorporating stabilizing agents in the formulation, use of surface coating technique, and appropriate control the storage conditions, such as temperature, pH, light and ionic strength of the storage medium to optimize the storage stability.

### In vivo UTI study

#### Urine analysis of WBCs

**Fig. 6:**
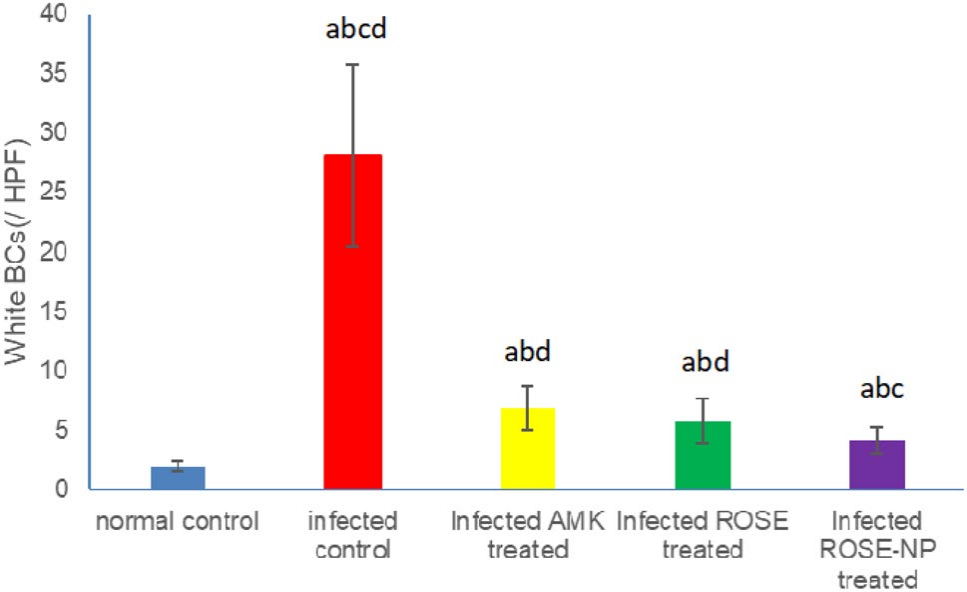
Effect of treatment with amikacin (AMK), rosemary oil (ROSE) and CS- Nanophytosomes on WBC’s count in the urine on infected rats (*n* = 6). **a**: significant difference compared to normal control. **b**: significant difference compared to infected control. **c**: significant difference compared to infected AMK treated group. **d**: significant difference compared to infected ROSE treated group (*p* < 0.001)

The untreated rats infected with UTI (Group II) exhibited a significant increase in WBC count (28.33 ± 4.12/HPF) compared to healthy rats (2.08 ± 0.81/HPF) (*P* < 0.001). Upon treatment, infected rats receiving either amikacin (Group III) or pure rosemary oil (Group IV) demonstrated substantial reductions in urine WBC count (6.83 ± 1.29/HPF and 5.83±/HPF, respectively). Interestingly, no statistical difference was observed between the WBC counts in these two treatment groups (*p* > 0.05), suggesting that rosemary oil has a similar effect in reducing WBC count as amikacin. Notably, the most significant reduction in WBCs occurred in Group V (4.17 ± 0.91/HPF), where rats were treated with CS nanophytosomes, compared to Group II (*P* < 0.001). The WBC count was significantly reduced by CS nanophytosomes in comparison to either Group III or Group IV (*p* < 0.05). This finding highlights the enhanced antibacterial effect of rosemary when delivered via CS nanophytosomes (Fig. 6). CS nanophytosomes effectively interacted with cell membranes, disrupting their structure and function, leading to leakage of cellular components [55].

#### Biochemical analysis

We investigated the impact of different treatments on key biochemical markers associated with UTI. Table 6 presents a comprehensive overview of the urea, creatinine, CRP, MDA, TAC, and IL-10 levels across the five groups. Compared to Group I, Group II rats exhibited elevated serum levels of both urea and creatinine, indicating compromised kidney function due to the infection. All treatment cohorts (Groups III-V) showed a significant reduction in creatinine levels compared to Group II (*p* < 0.001). However, amikacin (Group III), a positive control antibiotic, did not significantly improve urea levels compared to Group II (*p* > 0.05). Amikacin is known to have nephrotoxic effects, as it remains unmetabolized in the body, leading to its accumulation in the proximal tubule upon excretion in urine, where it generates free radicals causing renal damage [56]. Conversely, rosemary oil, particularly when delivered via CS nanophytosomes (Group V), normalized the serum concentrations of both urea and creatinine, underscoring its significant nephroprotective attributes. This observation aligns with the findings reported by Abdel-Azeem AS et al. [57], suggesting that rosemary oil may modulate intracellular pathways associated with DNA repair to ameliorate glomerular function and mitigate renal injury. Furthermore, rosemary oil has demonstrated the ability to activate the Nrf2/HO-1 pathway, thereby upregulating the expression of various protective genes and bolstering the efficacy of the endogenous cellular antioxidant defense system [58]. Moreover, the administration of CS nanophytosomes further enhanced the restoration of kidney function to baseline levels subsequent to UTI.

**Table 6:** Comparison of urea, creatinine, CRP, MDA, TAC, and IL-10 levels among all groups. (*n* = 6)

Groups	Urea (mg/dL)	Creatinine (mg/dL)	CRP (µg/mL)	MDA (nmol/mg protein)	TAC (µm/mg protein)	IL-10 (pg/mg protein)
I	41.16 ± 2.11	0.56 ± 0.01	121.0 ± 2.16	1.33 ± 0.11	53.1 ± 2.26	122.5 ± 4.71
II	71.83 ± 4.52	0.66 ± 0.01	179.5 ± 5.37	2.73 ± 0.35	31.8 ± 2.54	81.3 ± 7.78
III	72.33 ± 3.85	0.56 ± 0.01	166.5 ± 3.04	1.91 ± 0.18	40.6 ± 4.38	93.8 ± 4.45
IV	55.33 ± 2.42	0.55 ± 0.04	165.3 ± 2.56	1.62 ± 0.04	40.6 ± 1.97	97.1 ± 5.89
V	36.50 ± 2.62	0.53 ± 0.01	145.5 ± 5.79	1.46 ± 0.09	49.5 ± 3.86	114.0 ± 3.74

Reactive oxygen species (ROS) play a vital role in various biological processes, including apoptosis, immunity, and defense mechanisms against pathogens. However, elevated ROS levels can lead to cellular damage and oxidative stress (OS). Antioxidants sourced from endogenous or exogenous origins can act as protective shields and mitigate the detrimental impacts of OS [59]. Moreover, there exists a connection between oxidative stress (OS) and inflammation. These two processes are closely linked, often influencing each other. The connection between OS and inflammation involves a positive feedback loop: inflammation generates ROS, which, in turn, exacerbate inflammation. In general, OS can be evaluated by testing MDA levels, the primary marker of lipid peroxidation caused by ROS, and TAC levels which reflects the body’s ability to counteract ROS [60].

Infection led to an increase in serum MDA and a decrease in TAC levels were observed in Group II rats, indicating OS. In contrary, all treatment groups (Groups III-V) exhibited a significant decrease in MDA levels and an increase in TAC levels compared to Group II (*p* < 0.001), with Group V showing the most pronounced improvement, where TAC levels approached almost normal levels compared to Group I (*p* > 0.05). Amikacin and rosemary oil effectively alleviated OS through their antibacterial and anti-inflammatory properties. Rosemary oil, in particular, demonstrated a more potent antioxidant effect than Amikacin, further enhanced when delivered via CS nanophytosomes. Rosemary oil contains active phytochemicals such as flavonoids, polyphenols, and diterpenes, with strong antioxidant properties. These phytochemicals exhibite antioxidant capabilities by electron donation to reactive radicals, thereby reducing their reactivity, enhancing stability, and minimizing interactions with vital biomolecules such as DNA, lipoproteins, and polyunsaturated fatty acids [61].

Furthermore, the elevation of CRP and IL-10 levels are used to assess the progression of inflammation. UTI often results in inflammation as a response to bacterial invasion, leading to inflammation. When bacteria infiltrate the body, macrophages release cytokines like IL-1β, IL-6, and TNF-α. These cytokines initiate a series of inflammatory cascade through autocrine and paracrine mechanisms. Although this response aims to eliminate the infection, it can also result in substantial harm to the host tissue [62].

CRP is an acute-phase inflammatory protein that becomes elevated during various inflammatory conditions, including cardiovascular diseases, rheumatoid arthritis, and infections [63]. On the other hand, IL-10 is an anti-inflammatory cytokine which plays a crucial role in regulating inflammation during UTIs. In this study, untreated UTI rats (Group II) showed a significant increase in CRP levels and a decrease in IL-10 levels compared to those in normal control rats (Group I) (*p* < 0.001). In contrast, all treatment groups (Groups III-V) demonstrated a significant decrease in CRP levels and an increase in IL-10 levels, with Group V treated with CS nanophytosomes exhibiting the most remarkable improvement compared to the untreated rats in Group II (*p* < 0.001).

The antibacterial effect of both amikacin and rosemary oil leads to alleviation of inflammation, however, rosemary oil has additional anti-inflammatory mechanisms that have been documented [64]. It exerts anti-inflammatory activity through the reduction of the transcription factor NK-κB, hindering the pro-inflammatory mediators, such as TNF-α, IL-1β, synthesis and inhibiting synthesis of COX-2 enzyme, leading to decrease of arachidonic acid-metabolites downstream production [65]. The ability of rosemary oil to neutralize the reactive species generated during inflammation by its anti-oxidant properties may also mitigate damage caused by inflammation [66]. Furthermore, the anti-inflammatory effect was enhanced when rosemary oil was delivered via nanophytosomes due to improved bioavailability.

### Histopathological examination

**Fig. 7:**
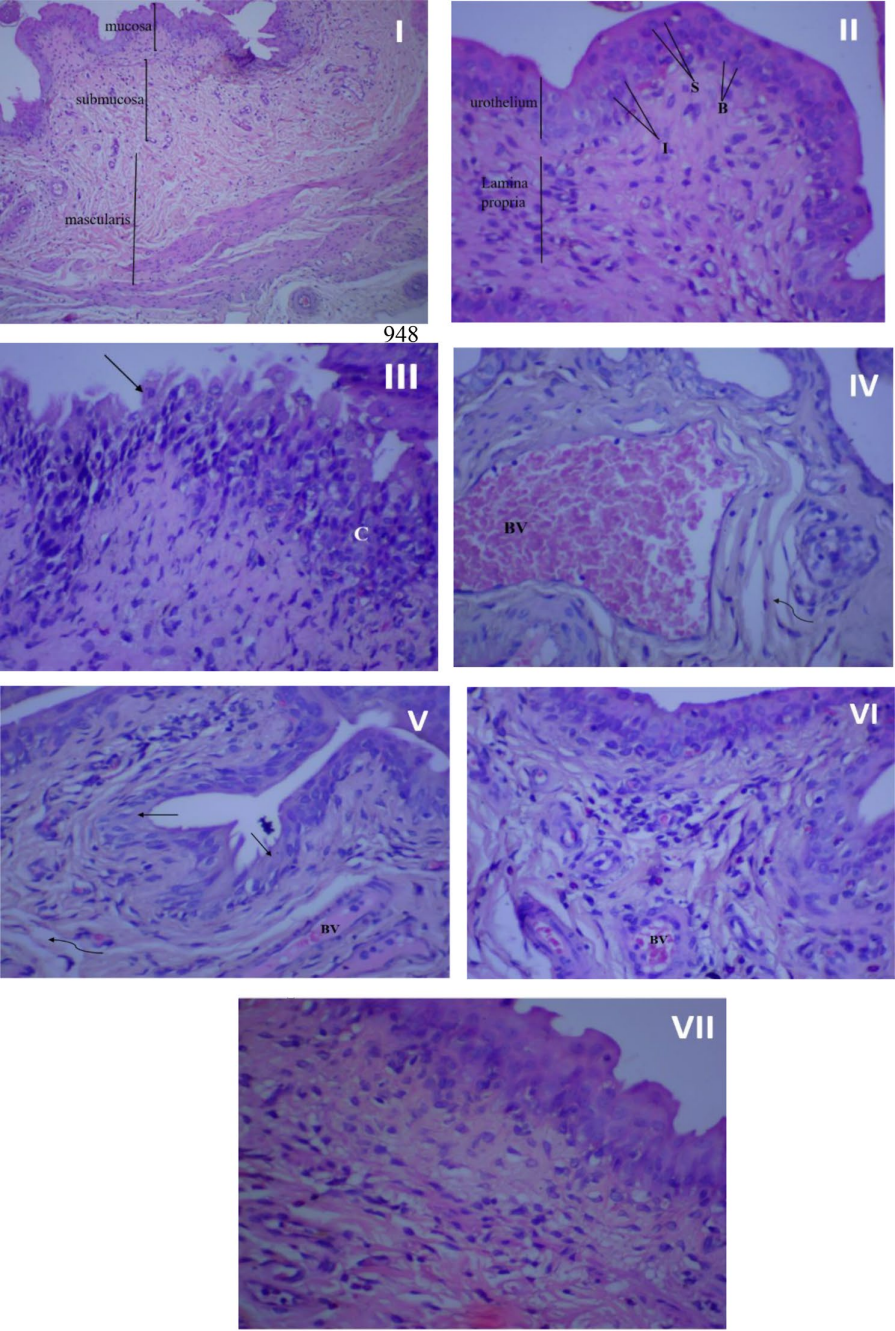
Histopathological examination of rat’s urothelial mucosa. (I) Normal bladder tissue (no infection and no treatment), x100 magnification. (II) Normal bladder tissue, x 400 magnification. (III) Bladder urothelium in the infected control group with chronic inflammation (C) in lamina propria, and urothelium desquamation (↑), x 400 magnification. (IV) Bladder urothelium in the infected control group showing dilated, congested blood vessel (BV), and mild fibrosis (wavy arrow). (V) Urinary bladder of rat treated with amikacin showing moderate inflammation in lamina propria, desquamation of urothelium (↑), mild fibrosis (wavy arrow) and congested blood vessels (BV), x 400 magnification. (VI) urinary bladder of rosemary oil treated rat showing many inflammatory cells beneath to urothelium, small congested blood vessel (BV) and Intact urothelium, x 400 magnification. (VII) urinary bladder of CS nanophytosomes treated rat showing significant improvement with well-defined urinary bladder architecture and some inflammatory cells in lamina propria, x 400 magnification

Figure 7 illustrates the histopathological findings of the rat urothelial mucosa. The bladder tissue of rats in the control groups (I, II) appeared normal, devoid of hyperplasia or inflammatory cells. However, following infection, the bladder tissues suffered significant damage, characterized by necrosis, urothelium desquamation, inflammatory cell infiltration, fibrosis, and congested blood vessels (III, IV). Treatment with amikacin (V), rosemary oil (VI), or CS-R nanophytosomes (VII) mitigated these pathological changes. Among these treatments, the CS-R nanophytosome-treated rats exhibited the most remarkable improvement, displaying a significantly enhanced and well-defined urinary bladder architecture.

**Fig. 8:**
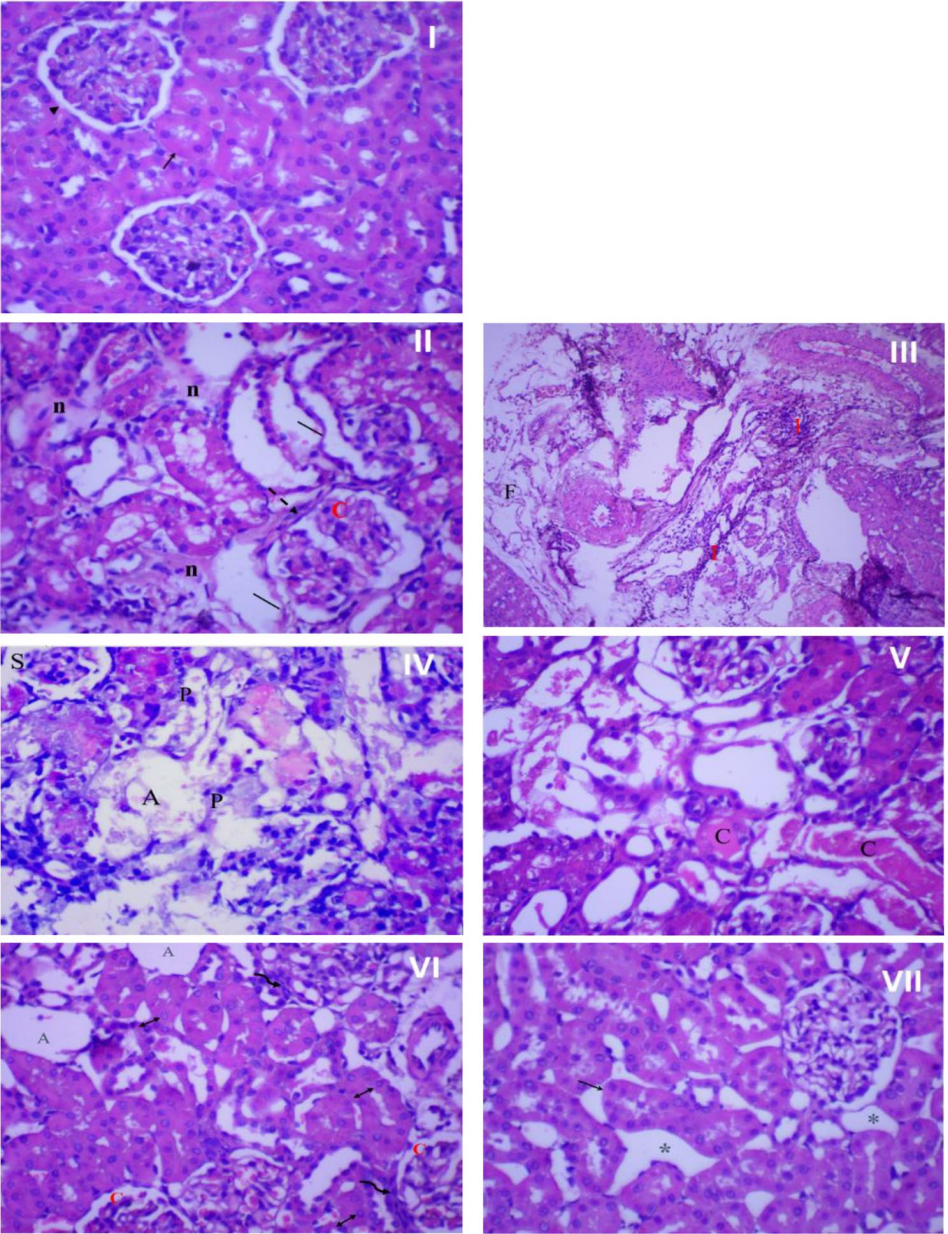
Histopathological examination of rat’s kidney. (I) Normal kidney tissue (no infection and no treatment) showing normal kidney architecture of proximal tubules (↑) with normal epithelial lining, and glomerulus (►), x 400 magnification. (II) Kidney in the infected control group showing severe damage with loss of kidney architecture, mild necrosis (n), congested glomerulus (C) with thick glomerulus wall (dash arrow), and enlarged, disorganized, desquamated (│) renal tubules, x 400 magnification. (III) Kidney in the infected control group showing severe inflammation (I) and fibrosis (F), x 100 magnification. (IV) Kidney of rat treated with amikacin showing damage and disorganization of kidney tubules, shrank (S), and atrophied (A) glomeruli. Most cells showing pyknotic (P) nuclei, x 400 magnification. (V) kidney of rat treated with amikacin showing severe and widespread necrosis of tubular epithelial cells. Most cells lose its architecture with cast (C) formation, x 400 magnification. (VI) kidney of rosemary oil treated rat showing atrophied (A) and congested (C) glomeruli surrounded by inflammatory cells (wavy arrow) and tubular dilatation with vesiculated nuclei (↕), x 400 magnification. (VII) kidney of CS nanophytosomes treated rat showing dramatic improvement, well-defined renal tubules with mild dilatation (↑) and spacing (*) between tubules, x 400 magnification

Figure 8 presents the histopathological examination results of rat kidney tissue. In the control group (I), the kidney tissue showed a normal structure with a healthy epithelial lining. However, infection resulted in significant kidney tissue damage characterized by necrosis, architectural loss, and infiltration of inflammatory cells (II), accompanied by fibrosis and congested blood vessels (III). Interestingly, the impact of amikacin treatment on kidney tissue differed from its effect on bladder tissue. In the kidneys, the renal tubules were damaged, shrank, and atrophied, with pyknotic nuclei in most cells (IV, V). However, treatment with rosemary oil (VI) or CS nanophytosomes (VII) alleviated these pathological changes. The most substantial improvement was observed in rats treated with CS nanophytosomes (VII). These rats exhibited a dramatic improvement with well-defined renal tubules showing mild dilatation and spacing between them. The improved architecture of both the kidney and bladder, observed in CS nanophytosomes, supports their antibacterial, anti-inflammatory, and nephroprotective effects, as demonstrated in our study.

## Conclusion

The investigation into environmental-friendly nanophytosomes addresses global concerns about the environmental impact of pharmaceutical products. The studied CS nanophytosomes combine the biodegradable properties of chitosan with the eco-friendly attributes of rosemary. Chitosan, a natural polymer, naturally decomposes through microbial processes, making it an environmentally friendly matrix [56]. Additionally, rosemary oil is encapsulated within chitosan nanoparticles to prevent volatile losses and undesirable changes. As these nanophytosomes degrade, they gradually release the encapsulated rosemary oil, which can be harmlessly metabolized, minimizing ecological impact [57]. Both chitosan and rosemary oil align with eco-friendly principles, being non-toxic, biocompatible, and supportive of sustainable practices.

This study demonstrates that CS nanophytosomes encapsulating rosemary oil provide a sustainable and cost-effective approach to combat difficulty-to-treat urinary tract infections (UTIs) caused by multidrug-resistant (MDR) *E. coli*. The antibiofilm activity and the antioxidant and anti-inflammatory properties of rosemary oil are enhanced when delivered via CS nanophytosomes, as they facilitate its delivery and increase its bioavailability. Furthermore, the CS-R nanophytosomes improve histopathological damage in bladder and kidney tissues associated with *E. coli* UTIs and exert a nephroprotective effect; therefore, they hold promise as a sustainable alternative therapeutic option for MDR *E. coli*-induced UTIs. Nevertheless, the limitation identified is the need for further research to optimize the long-term storage stability of nanophytosomes under varying conditions to ensure consistent quality during scale-up manufacturing for clinical use. For future applications of CS-R nanophytosomes, it is essential to address the challenging aspects of standardization, regulatory requirements, and bioavailability.

## Electronic supplementary material

Below is the link to the electronic supplementary material.

Supplementary Material 1

## Data Availability

No datasets were generated or analysed during the current study.

## Abbreviations

CFU: Colony-forming unit
CRP: C-reactive protein
CS: Chitosan
EE: Entrapment efficiency
EO: Essential oil
FTIR: Fourier transform infrared
i.p.: Intraperitoneal
IL-10: Interleukin-10
MDA: Malondialdehyde
MDR: Multidrug-resistant
MIC: Minimum inhibitory concentration
NSLC: Nanostructured lipid carrier
OD: Optical density
OS: Oxidative stress
PDI: Polydispersity index
PS: Particle size
ROS: Reactive oxygen species
TAC: Total antioxidant capacity
TEM: Transmission electron microscopy
TPP: Tripolyphosphate
TSB: Tryptic soy broth
UTI: Urinary tract infection
ZP: Zeta potential
